# Gefitinib Results in Robust Host-Directed Immunity Against *Salmonella* Infection Through Proteo-Metabolomic Reprogramming

**DOI:** 10.3389/fimmu.2021.648710

**Published:** 2021-03-31

**Authors:** Srikanth Sadhu, Zaigham Abbas Rizvi, Ramendra Pati Pandey, Rajdeep Dalal, Deepak Kumar Rathore, Bhoj Kumar, Manitosh Pandey, Yashwant Kumar, Renu Goel, Tushar K. Maiti, Atul Kumar Johri, Ashutosh Tiwari, Amit Kumar Pandey, Amit Awasthi

**Affiliations:** ^1^ Infection and Immunobiology, Translational Health Science and Technology Institute, Faridabad, India; ^2^ Department of Biotechnology and Biochemistry, SRM University, Sonepat, India; ^3^ Infection and Immunity, Translational Health Science and Technology Institute, Faridabad, India; ^4^ Functional Proteomics Laboratory, Regional Centre for Biotechnology, Faridabad, India; ^5^ Non Communicable Diseases, Translational Health Science and Technology Institute, Faridabad, India; ^6^ Infection and Immunity, Jawaharlal Nehru University, New Delhi, India

**Keywords:** *Salmonella*, gefitinib, HIF-1α, mTOR, EGFR, acriflavin, host directed therapeutics, proteo-metabolomic changes

## Abstract

The global rise of antibiotic-resistant strains of *Salmonella* has necessitated the development of alternative therapeutic strategies. Recent studies have shown that targeting host factors may provide an alternative approach for the treatment of intracellular pathogens. Host-directed therapy (HDT) modulates host cellular factors that are essential to support the replication of the intracellular pathogens. In the current study, we identified Gefitinib as a potential host directed therapeutic drug against *Salmonella*. Further, using the proteome analysis of *Salmonella*-infected macrophages, we identified EGFR, a host factor, promoting intracellular survival of *Salmonella via* mTOR-HIF-1α axis. Blocking of EGFR, mTOR or HIF-1α inhibits the intracellular survival of *Salmonella* within the macrophages and in mice. Global proteo-metabolomics profiling indicated the upregulation of host factors predominantly associated with ATP turn over, glycolysis, urea cycle, which ultimately promote the activation of EGFR-HIF1α signaling upon infection. Importantly, inhibition of EGFR and HIF1α restored both proteomics and metabolomics changes caused by *Salmonella* infection. Taken together, this study identifies Gefitinib as a host directed drug that holds potential translational values against *Salmonella* infection and might be useful for the treatment of other intracellular infections.

## Introduction

Typhoidal and non-typhoidal fever caused by *Salmonella* infection is a global health challenge with an estimated annual mortality of 128000-161000 (1–3). *Salmonella* infects the host through the intake of contaminated food or water and crosses the intestinal epithelial barrier causing inflammatory response (3, 4). Like other intracellular pathogens, *Salmonella* is phagocytosed by macrophages, monocytes, neutrophils, and subsequently leads to localized gastrointestinal or systemic infections that are characterized by diarrhea (3–6). However, therapeutic approaches against *Salmonella* have long relied upon the use of antibiotics with high success (3, 4, 7).The emergence of antibiotic-resistant strains (8) posing an urgent and emerging need to develop the novel therapeutic strategies. In addition to antibiotics, new approaches involve the development and faster testing of vaccines against *Salmonella* using a controlled human infection model (9). Nonetheless, the emergence of HDT (Host Directed Therapeutics) provides an alternative and attractive strategy to treat sporadic *Salmonella* infection (10–12), and it is becoming increasingly popular because it offers effective targeting of bacterial multi-drug resistance strains with cost-effectiveness. HDT involves designing of novel drug candidates after the identification of host factors that are being utilized and modulated for their survival by the pathogens. These factors, such as signaling molecules (calcium signaling and G protein-coupled receptors), and membrane cholesterol distribution enzymes of essential metabolic pathways, transcription factors or immune checkpoints, make attractive HDT targets since these are exploited by the pathogens to support their replication within the host cell (10, 11). These pathways play an essential role in adhesion, entry and replication of bacteria’s inside the host cell. Therefore, we screened ENZO library of small molecules which targets these processes in the host cell. The screening of the library results in identification of compounds that interfere with the host molecules to suppress the replication of the salmonella with in the host cell (11).

The infection of intracellular pathogens is accompanied by rapid metabolite changes within the host as pathogen divides and multiplies by utilizing the host machinery (13–15). It is now well established that multiplication of these intracellular bacteria may induce hypoxia resulting in the Warburg effect to meet energy demands (14–17). Interestingly, induced low oxygen burden in the host cells resulting in hypoxia has also been reported in *Salmonella* infection (16–18). In response to a hypoxic condition, infected host cells express HIF (Hypoxia induced Factor), the master regulators sensing low oxygen levels (16, 18). HIF consists of a constitutively active β-subunit and an oxygen labile α-subunit, which exists in three forms termed HIF-1α, -2α and -3α. HIF-1α is known to be expressed by all the immune cells, including macrophages, and is central to the response against hypoxia (16, 19–21). HIF-1α protein is induced and stabilized under low oxygen conditions and leads to shifting of the balance from aerobic oxidation of glucose to anaerobic oxidation (19). Furthermore, during infection, hypoxia is often accompanied by Warburg effects resulting in metabolic reprogramming, which involves switching to anaerobic oxidation from the Krebs cycle with subsequent metabolism of glucose to lactate (13, 22, 23). This results in a high rate of glycolysis to produce biosynthetic precursors, which provides building blocks for cellular macromolecules during its rapid multiplication inside the host cell.

In the present study, we establish, using compounds library screening and proteome analysis of uninfected and *Salmonella*-infected THP1 cells that utilizes EGFR-HIF-1α axis to promote its replication within the macrophages. EGFR inhibitor, Gefitinib, suppresses the replication of *Salmonella* by suppression of EGFR signaling within macrophages. Furthermore, *in vivo* treatment of *Salmonella*-infected mice with Gefitinib showed remarkable recovery associated with enhanced Th1 response and decreased frequency of myeloid-derived suppressor cell (MDSC). Moreover, inhibition of EGFR signaling induces proteo-metabolomics reprogramming that mitigates the effects of *Salmonella* infection. Taken together, we report that blocking of the EGFR-HIF1α axis may act as a potent HDT, which may have translational potential in targeting drug-resistant *Salmonella* infection.

## Materials and Methods

### Mice

FVB mice were obtained from National Institute of Immunology, New Delhi, India (Small animal facility) and maintained at THSTI (small animal facility) under the condition of 12 h light/dark cycle with *Ad libitum* chow and water supply. All animals used for the experimental purpose were of age group 6-8 weeks and were approved by the institutional IAEC Committee.

### Reagents

Acriflavine (A8251), Rapamycin (SML 1657), EGF (Sigma RP 3027), p-EGFR (CST 2234S), p-S6 (CST 4858S), ATP (Sigma A2383), Suramin (Sigma S2671), 2-DG (Sigma D8375), Gefitinib, PMA were obtained from Sigma Aldrich (US). Reagents for ELISA, including anti-mouse IFNγ, anti-mouse TNFα, anti-mouse IL-17A, anti-mouse IL-10 (purified and biotinylated) were obtained from Biolegend (California). Western blot antibodies, anti-human HIF1α, anti-human mTOR, anti-human p70-S6, and anti-human β-actin were purchased from CST (US). Purified anti-mouse HIF1α antibody for immunohistochemistry was purchased from R&D systems (US). Anti-mouse CD3 BV510, anti-mouse γδTCR FITC, anti-mouse Gr1 BV421, anti-mouse CD11b PerCp-Cy5.5 for immunophenotyping were purchased from BioLegend (California). Gentamycin, SS agar (Hi media M108D), LB media, RPMI-1640 (Sigma AL060A; 500ml), L-glutamine (Invitrogen A2916801), Pen/Strep (Thermo Scientific 11360070), Tween 20 (Sigma 9005-64-5), ENZO compound library.

### *Salmonella* Strains and THP-1 Cell Line

S. Typhimurium (SL 1344) and luciferase-expressing Xen33 (FDA 1189) strains were a kind gifted by C. V. Srikanth (RCB) while *S*. *typhi* (Vi+) was gifted by A. Sharma (THSTI). Bacteria were cultured until the log phase and then sub-cultured in LB media with a (1:50 dilution) for 3 h at 37°C with constant shaking. The optical density of the culture was then taken at 600 nm for a quick estimation of CFU (1 OD_600nm_= 1x10^8^CFU/ml).

THP-1 cell line was purchased from ATCC (Rockville, MD, USA) and cultured in DMEM, reconstituted with 10% heat-inactivated FBS (Gibco 10082147) and 55µM β-mercaptoethanol (Sigma M6250) in 5% CO_2_ incubator at 37°C.

### *Salmonella* Infection and Compound Screening

For establishing infection, 0.04 million THP-1 cells were seeded in 96-well plates per well and cultured in the presence of PMA (50ng/ml) for24 h at 37°C. An infection of 1:10 MOI was then established in the wells containing activated THP-1 cells and co-incubated at 37°C in 5% CO_2_ incubator. Treatments of inhibitors/modulators were given post-infection for 24 h to determine the intracellular survival rate. For assessing the inhibition of internalization by compounds, the cells were pre-treated, washed with PBS, and then infected with *Salmonella* (For 30min.), further incubated in RPMI containing 100 µg/ml gentamicin for 1 h at 37°C followed by an RPMI wash. The infected THP-1 cells were then used directly or lysed in 0.1% Triton X-100. From the lysate of the THP-1 cells, colony plating was carried out for *Salmonella* on SS agar plates. The number of colonies was counted manually. The viability of THP-1 cells was determined by using a trypan blue exclusion assay by using a hemocytometer under a microscope.

### Direct Killing Assay

The direct killing assay was performed using Presto blue dye (Thermo Scientific) in a colorimetry based assay. Briefly, the compounds were added directly to *S. typhi* cells in a 96 well plate. The cells and the compounds were co-incubated in a 5% CO_2_ incubator for 24 h. After 24 h incubation, 20µl of Presto blue (Thermo scientific) was added directly to the culture media, and changes in color were determined through colorimetry.

### Establishment of *Salmonella* Infection Model in BMDMs

Femurs and tibia were collected from sacrificed C57BL/6 mice. These bones were then cleaned and flushed from one side in a culture dish. The cells obtained were centrifuged and cultured in DMEM containing 10% FBS and 50 ng/ml M-CSF (BD Biosciences) for 5-6 days, and purity was analyzed by Flow cytometry. These differentiated macrophages were used for further *Salmonella* infection experiments.

### Hypoxia Study

THP1 differentiated macrophages were infected with *Salmonella* with 1:10 MOI (Cells: Bacteria) and were cultured in presence or absence of Rapamycin (50nM), Gefitinib (1µM, 5µM, 10µM) or Acriflavin (5µM; HIF1α inhibitor). These cells were incubated either in 5% CO_2_ incubator or in an artificial hypoxia chamber (1% O_2_) for 24h. Thereafter, the cultured cells were washed twice with PBS. The cells were then lysed with 0.1% Triton X-100 and the diluted lysate was then spread on SS agar plates to enumerate viable CFU count.

### mRNA Expression Profiling Through qPCR

RNA isolation by RNAeasy kit (Qiagen) followed by cDNA synthesis (BIO-RAD) was carried out as per the manufacturer’s manual. Real-time PCR was performed as described by Malik et al. (24) by using SYBR green dye KAPA SYBR FAST qPCR Master Mix (2X) Universal kit (KK 4600) on Fast 7500 Dx real-time PCR system (Applied biosystems) (24). Obtained results were analyzed by SDS2.1 software. The cycling threshold value of the endogenous control genes β-actin (for mouse) and GAPDH (for Human) was subtracted from the cycling threshold value of each target gene to generate the change in the cycling threshold (ΔCT). The relative expression of each target gene was expressed as “fold change” relative to that of respective samples (2ΔCT). We used the previously used formula (POWER (2,-ΔCT)*10000 to calculate the relative gene expression (24, 25). The acquisition of qPCR was done on Applied Biosystems 7500 RT-PCR systems. The sets of primers (forward and reverse respectively) used for the THP-1 cells were GAPDH (5’-ACAGTTGCCATGTAGACC-3’;

5’-TTGAGCACAGGGTACTTTA-3’), HIF1α (5’-AAAATCTCATCCAAGAAGCC-3’

5’-AATGTTCCAATTCCTACTGC-3’), Glut1 (5’CAGTTCGGCTATAACACTGGTG-3’

5’-GCCCCCGACAGAGAAGATG-3), HK2 (5’-TGATCGCCTGCTTATTCACGG-3’

5’-AACCGCCTAGAAATCTCCAGA-3’), LDH5 (5’-CATTGTCAAGTACAGTCCACACT-3’ 5’-TTCCAATTACTCGGTTTTTGGGA-3’), PKM (5’-GCCGCCTGGACATTGACTC-3’ 5’-CCATGAGAGAAATTCAGCCGAG-3’), MCT4 (5’-TCACGGGTTTCTCCTACGC-3’ 5’-GCCAAAGCGGTTCACACAC-3’), TPI (5’-CCAGGAAGTTCTTCGTTGGGG-3’ 5’-CAAAGTCGATGTAAGCGGTGG-3′), Eno 1 (5’-TGCGTCCACTGGCATCTAC-3’ 5’-CAGAGCAGGCGCAATAGTTTTA-3’). For studies involving mouse samples following sets of primers were used: β-actin (5’-GATGTATGAAGGCTTTGGTC-3’ 5’-TGTGCACTTTTATTGGTCTC-3’), IFNγ (5’-TGAGTATTGCCAAGTTTGAG-3’ 5’-CTTATTGGGACAATCTCTTCC-3’), HIF-1α (5’- CGATGACACAGAAACTGAAG-3’ 5’-GAAGGTAAAGGAGACATTGC-3’), IL-10 (5’-CAGGACTTTAAGGGTTACTTG-3’ 5’-ATTTTCACAGGGGAGAAATC-3’), EGFR (5’-TGACTACTACGAAGTGGAAG-3’ 5’-CATTACAAACTTTGCGACAG-3’).

### Western Blotting

Cell samples were washed with PBS and protein extraction was carried out by RIPA buffers. The concentration of protein in each sample was determined by performing BCA protein assay (Bio-Rad). 50 µg of protein extracts were loaded on a 10% Tris-tricine gel in an MES buffer (Invitrogen) or 3–8% Tris-glycine gel in a Tris-acetate buffer (Invitrogen) for Mtor (CST 2972S), p70-S6, HIF-1α, P-EGFR, P-s6, β-actin respectively. After electrophoresis, proteins were transferred to a nitrocellulose membrane; the membrane was blocked in 5% non-fat milk and then incubated in primary antibody diluted in 5% non-fat milk overnight at 4°C. The peroxidase-conjugated goat anti-rabbit (BD Biosciences) secondary antibody was incubated further for 1 h. Immunoreactive proteins were detected using the ECL chemiluminescent system (BIO-RAD) and bands were captured on Gel-doc (BIO-RAD). Band intensities for each western blot were calculated by using ImageJ software.

### Nucleofection Protocol

Cultured THP-1 cells were seeded 1.5x10^7^ in a 75cm^2^ flask and activated with 10ng/ml L-Glutamine and 50µM β-mercaptoethanol for 48 h. Cells were then treated with accutaseI and kept at 37°C incubator for 30 min. Healthy cells as confirmed under the microscope were pelleted down at 300xg for 15 min. 0.2x10^6^ THP-1 cells were then suspended in 100µl Amaxa™ 4D-Nucleofector Solution (LONZA; V4XP-3024) and nucleofected with HIF1α gRNA plasmid on 4D-Nucleofector™ X unit (Lonza) using THP-1 Cell 4D-Nucleofector™ X Kit (Lonza, V4XP-3024) as per manufacturers protocol. Nucleofection was confirmed by western and flow cytometry and was used for further experiments.

### *Salmonella* Infection in the Mice

The gastroenteritis model for *Salmonella* infection was established through oral gavage administration. Briefly, FVB mice were starved of food and water for 4 h and then orally gavaged with 7.5 mg streptomycin to sterile the gut. After 20h mice were again starved of food and water for 4 h. Overnight culture of *Salmonella* and further sub-cultured for 4h were infected in these mice at 1x10^6^ CFU by oral gavage. After the successful establishment of infection, mice received either Gefitinib (80mg/kg every alternate day) (26, 27) or Acriflavin (5mg/kg each day). At the end of the study, the mice were sacrificed and their tissues and organs were utilized for molecular and histological studies.

### Histology and Immunohistochemistry Studies

FVB mice were infected with S. Typhimurium and received Gefitinib or Acriflavin as described above. The mice were sacrificed at day 7 and their intestine, caecum was removed and used further for histological studies. The organs were washed with PBS and fixed in 10% formaldehyde for overnight. Tissue blocks were prepared on Paraffin wax and thereafter the organs were fine-sectioned on microtome and hematoxylin and eosin (HE) staining was carried out. The HE stained sections were washed and rat anti-mouse hypoxia-inducible factor- 1α antibody was exposed to the sections followed by a secondary antibody. The finally stained sections for HIF1α were then observed and images were captured under The Eclipse Nikon microscope. The quantitation of staining was done by random scoring by unbiased unrelated observers.

### Immunophenotyping

On 7^th^ day post-infection the mice were sacrificed and their spleen and MLN were isolated. Single-cell suspension prepared and stained (1 x 10^6^ cells) with fluorescently labeled anti CD3 (BD Biosciences USA), anti-mouse CD4 (BD Biosciences USA), anti-mouse γδ-TCR (eBioscience Invitrogen, USA), CD11b (eBioscience Invitrogen, USA) or Gr1 antibody (eBioscience Invitrogen, USA) for 20 min at RT followed by twice washing with PBS. Stained samples were then acquired on BD FACS Canto (BD Bioscience) and analyzed with the help of FlowJo software (Vx 0.7 Treestar).

### *In Vivo* Imaging for Therapeutic Efficacy

FVB mice were infected orally with Xen-33 *Typhimurium* and its bioluminescence was measured on 5^th^ day post-infection. Mice were anesthetized with 2% Isoflurane for bioluminescence *in vivo* imaging with IVIS Lumina (Perkin Elmer) and quantified with living image software version 4.2. Bioluminescent levels were measured as radiance (p/sec/cm^2^/sr), by using a pseudo scale for measurement in which blue represents low intensity, and red represents high intensity. Posts *in vivo* imaging, the bacterial burden in stool sample cultures were also measured.

### ELISA

Sandwich ELISA was performed for the quantitation of IFN-γ, TNF-α, IL-17, IL-10 cytokines obtained from mouse serum samples by using their respective ELISA antibodies (Biolegend). The absolute quantitation of the cytokines (in pg/ml) was done by using individual standard recombinant cytokines. ELISA was performed by a coating of the primary antibody to the walls of the flat bottom 96 well plate. Thereafter, the unbound antibody was washed with PBS + 0.05% tween20. Blocking was done with a 3% FBS solution at 4°C overnight followed by biotin-conjugated secondary antibody incubation for 1 h at room temperature. HRP-peroxidase conjugated with streptavidin was used to label the antigen bound secondary antibody complex. TMB (BD Biosciences) substrate (50µl/well) was then added to each well and incubated for 10-20 min, and the reaction was stopped by 0.2N HNO_3_ solution. The changes in the color intensity were measured through colorimetry at 540nm.

### Bacterial Burden in Mouse Feces

Group of 6 FVB mice of 8 weeks old were infected orally (1x10^6^CFU). The inoculum was prepared from a log phase culture of 0.7- 0.8 OD. This was grown at 37°C on a shaker in standard broth with kanamycin. Cells were washed and re-suspended in sterile PBS and administered orally by a 1 mm ball-tipped gavage needle. Feces were collected at the peak of infection and plated on the SS agar plate for determining its CFU.

### Metabolomics Profiling by LC-MS/MS

Serum from FVB mice was isolated, and protein precipitation was carried out with the help of LC-MS grade methanol 1:3 ratio (Sample: methanol) with continuous vortex for 30 sec followed by incubation on ice for 10 min. These samples were then centrifuged at 15000g for 20min at 4°C. The supernatant was collected, from this fraction (60μl) of the samples and transferred to sterile LC vials. These samples were kept for drying (mi Vac Duo Concentrator, Gene vac Ltd., U.K.), and the dry pellet was dissolved in 8:2 (v/v) acetonitrile: water for metabolomics analysis using chromatography system 5000 (Thermo Fisher). Polar metabolite dissociation attained with the NH2 column were further processed and analyzed.

All data were acquired on the orbitrap fusion mass spectrometer equipped with heated electrospray ionization (HESI) source. Data were acquired on positive and negative mode at 120,000 resolution in MS1 mode and 30000 resolution in data-dependent MS2 scan mode. We used a spray voltage of 4000 and 3500 volt for positive and negative modes respectively. Sheath gas and auxiliary gas were set to 42 and 11 respectively. The mass scan range was 60-900 m/z, AGC (Automatic gain control) target at 100000 ions and maximum injection time was 50 ms for MS and AGC target was 20000 ions and maximum injection time 60 ms for MSMS was used.

Extracted metabolites were separated on UPLC ultimate 3000 using XBridge BEH Amide column (100x2.1 mm i.d, 2.5 micrometers, Waters Corporation) maintained at 35 degrees C temperature. The mobile phase A was 20mM ammonium acetate in the water of PH 9.0 and mobile phase B was 100% acetonitrile. The elution gradient is used as follows: 0 min, 85% B, 2 min, 85%B, 12 min, 10%B, 14 min, 10%B, 14.1min, 85%B, and 16 min, 85%B. The flow rate of 0.35mL/min and the sample injection volume was 5 microliter.

### Data Processing

All acquired data has been processed using Progenesis QI for metabolomics (Waters Corporation) software using the default setting. The untargeted metabolomics workflow of Progenesis QI was used to perform retention time alignment, feature detection, deconvolution, and elemental composition prediction. Metascope plug of Progenesis QI has been used for the in-house library with accurate mass, fragmentation pattern and retention time for database search. We have also used online polar compounds spectral library for further confirmation of identification.

### Sample Preparation for Proteomic Study

1x10^6^ THP-1 cells were seeded in 6 well plates and activated with PMA (50ng/μl) for 48 h. Thereafter, *Salmonella* infection was established at 1:10 MOI in the presence or absence of Gefitinib or Acriflavin for 24 h. Cells were then washed with PBS, and their protein content was isolated by using RIPA buffer (Sigma) following the manufacturer protocol. Total protein concentration of cell lysate was determined using BCA™ protein assay kit (Thermo Scientific). Proteins from cell lysate was further subjected to in-solution digestion, 30 µg proteins were reduced (10 mM DTT for 1 h at 60°C), alkylated (20 mM IAA in dark at room temperature for 30 min) and then digested by trypsin (1:20 w/w in 50 mM TEAB) at 37°C for overnight. The digested sample was desalted and purified using C18 tip and further vacuum dried and stored at -20°C for mass spectrometry analysis For each condition three biological samples was prepared.

### Quantitative Proteomic Using Data-Independent Acquisition (DIA) Method

Digested peptides were analyzed on Triple- TOF 5600 (AB Sciex, Canada) mass spectrometer coupled with ekspert nanoLC400 (Eksigent, CA, USA) in high-sensitivity mode. Data were collected in SWATH acquisition mode and the instrument was configured as described by earlier (28) with slight modification. Briefly, a set of 45 overlapping (1 Da) windows were constructed covering the mass range 350–1250 Da. Peptide were separated using ChromXPcolumn (150×0.15mm, 3µm, 120 Å)with 125 min linear gradient of 2% to 40% mobile phase (Mobile phase A: 98% water, 2% acetonitrile with 0.1% (v/v) formic acid, mobile phase B: 98% acetonitrile with 0.1% (v/v) formic acid) at a flow rate of 250 nl/min. Fragment spectra were collected from 200 to 1600 m/z with 60 ms dwell time for fragment-ion scans resulting in a duty cycle of 3.4s. For SWATH processing and quantification, the raw file was processed in Spectronaut Pulsar (Biognosys) in the Direct-DIA method using default parameters, data were searched against UniProtKB protein database (contains 20069 human protein entries) includes iRT peptides (Biognosys). The FDR was set to 1% at the peptide precursor level. The extracted-ion peak intensities were further used for the quantitative analysis of proteins. We used Post Analysis Perspective feature of Spectronaut Pulsar (Biognosys) for further quantitative statistical analysis. Fold changes was expressed as log2 transformed ratios of mean value of three biological replicates. One-way analysis of variance (ANOVA) was used to compare natural log-transformed protein abundance values of control, infected, infected+ Gefitinib, infected+ Acriflavin, and infected+ Rapamycin samples. The proteins were considered to have changed significantly only if abundance changed > ± 1.5 fold with <0.05 corrected p-value (two multiple test correction method Bonferroni and Benjamini-Hochberg was applied).

### Functional enrichment analysis

The functions of differentially regulated proteins were analyzed using KEGG database, Network analysis was performed using Network Analyst (29) and DAVID (30) was used for pathway analysis. The intensities of regulated proteins were log (base 2) transformed and then used for volcano and heatmap for plot using the R software package.

### ATP Quantification

ATP levels were measured in the culture supernatants harvested from the THP-1 cells in different conditions using ATP determination kit (ThermoFisher; A22066) as per manufacturer instructions.

### Statistical Analyses

Each experiment was repeated at least three times independently and three biological replicates were used. Data were plotted as mean – SEM. Student’s t-test (Mann-Whitney), two way ANOVA and one way ANOVA (Kruskal-Wallis) were performed wherever applicable by using Graph Pad Prism 7.0 software. For the survival curve, Kaplan Meier statistical analysis was performed. P < 0.05 was considered to be statistically significant.

## Results

### EGFR Signaling Essential for the Survival of *Salmonella* Within Macrophages

**Figure 1:**
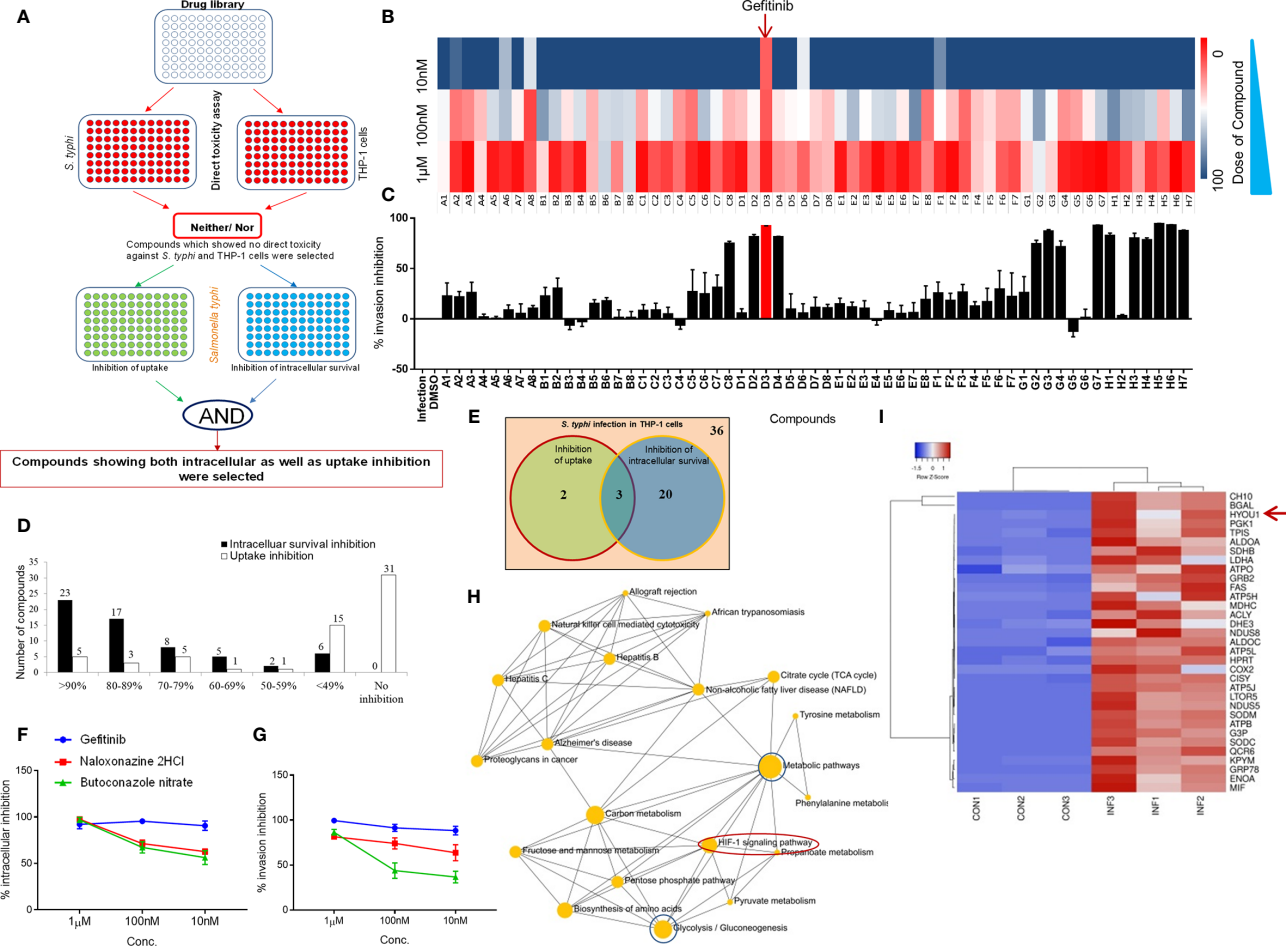
EGFR signaling essential for the survival of *Salmonella* within macrophages. Panel **(A)** representative strategy for the screening of ENZO compound library. **(B)** Intracellular replication as % inhibition was evaluated at different concentration (1µM, 100nM & 10nM) and plotted as intensity heatmap. Panel **(C)** represents % inhibition ± SEM upon pre-exposure of compounds (1µM) plotted as bar graphs. **(D)** The number of hits in the form of compounds showing good inhibition was plotted as a bar graph. **(E)** Venn diagram showing the number of compounds with ≥ 90% inhibition and 3 overlapping compounds with significant inhibition for both intracellular survival and internalization. These 3 compounds were further tested for intracellular and internalization inhibition in a dose kinetics assay (**F, G** respectively). **(H)** Network analysis of control and infection conditions. **(I)** Heatmap analysis of a subset of top significantly expressed genes in Control vs Infection. Data are representative of mean ± SEM from three replicates and each individual experiments repeated three times.

To understand the role of cholesterol distribution on plasma membrane, calcium signaling and G protein-coupled receptors in Salmonella-infected macrophages, an FDA approved ENZO library of small molecules (**Supplementary Table 1**) was screened to identify the inhibitors against intracellular replication of *Salmonella* within the macrophages. We measured internalization and intracellular replication of *S. typhi* in THP-1 cells, a prototypic human immature macrophage cell line, as shown in a schematic diagram **(** **Figure 1A** **)**. Library was first tested for its direct toxicity on *S. typhi* or THP-1 cells through Presto blue dye **(** **Supplementary Figure 1A** **)**. Interestingly, none of the small molecules of the library exerted any direct toxicity on *S. typhi* or THP-1-derived macrophages **(** **Supplementary Figure 1A** **)**. These compounds were then screened individually to evaluate their ability to inhibit the intracellular replication of *S. typhi* in PMA (Phorbol 12-myristate 13-acetate) -induced differentiated THP-1cells **(** **Supplementary Figure 1B** **)**. Further, dose-dependent kinetics for intracellular replication of *S. typhi* was performed for the compound library and it was observed that Gefitinib inhibits the intracellular replication of *S. typhi* within the macrophages even at low concentrations **(** **Figure 1B** **)**. We observed that 23 compounds of the library were showing >90% inhibition of *Salmonella* survival at 1.0 µM concentration, as we decrease the doses from 1.0 µM to 100nm and 10nm, the antimicrobial activity of small molecules reduces greatly (**Supplementary Figure 1C**). Interesting to note that Compound D3 (Gefitinib) of the small molecule library able to suppress intracellular replication of *S. typhi* even at the lower concentration (**Supplementary Figure 1C**). These small molecules were further screened to test their ability to inhibit the internalization of *S. typhi* by pre-treatment of the THP-1 cells with small molecules. For this purpose, THP-1 cells were incubated with library compounds and washed thereafter, followed by *S. typhi* infection. We found that 5 compounds showed ≥ 90%, 9 compounds showed>80%, 14 compounds >50% showed inhibition of *S. typhi* internalization (**Figure 1C**). We further combined the data of inhibition and internalization of S. *typhi* within the THP-1 cells, and found that 23 compounds of the library showed <90% inhibition of intracellular survival while 5 compounds showed <90% of inhibition of S. *typhi* uptake **(** **Figure 1D** **)**. We further selected the compounds that can suppress both intracellular survival and uptake of S *typhi* efficiently (<90%). Venn diagram indicated that 3 out of 61 compounds blocked intracellular survival and uptake of *S. typhi* **(** **Figure 1E** **)**. These three compounds, Gefitinib, Butaconozole nitrate, Naloxonazine 2HCl, were further tested for dose kinetics experiment to identify compound/s which can block both intracellular survival and uptake of S. *typhi* at lower concentrations. As indicated, among three compounds, Gefitinib potently inhibited both intracellular survival and uptake of *S. typhi* in THP-1 at lower concentrations **(** **Figures 1F, G** **)**, suggesting Gefitinib as a potential candidate that suppresses *Salmonella* infection. Since *Salmonella* also infects the epithelial lining of the gut, therefore we tested whether Gefitinib also suppresses *Salmonella* replication in Caco2 cells, a prototype intestinal epithelial cell. We found that similar to macrophages, Gefitinib also suppresses the intracellular survival of *S. typhi* in Caco-2cells **(** **Supplementary Figure 1E, F** **)**.

Gefitinib is an EGFR inhibitor, which suppress EGFR phosphorylation reversibly and further block EGFR signaling (31). Next, to identify the downstream of the EGFR signaling pathway, which supports *Salmonella* survival within the macrophages, we analyzed a subset of the proteome to understand the pathways which are upregulated in *Salmonella*-infected THP-1. We found that the HIF-1α signaling pathway was enriched in *S. typhi* infected THP-1 as compared to uninfected THP-1 cells (**Figure 1H**). Our proteome analysis also finds the upregulation of HYOU1 (Hypoxia upregulated protein 1) protein in S. *typhi*-infected THP-1 cells (**Figure 1I**). Altogether, these data demonstrated that EGFR and Hypoxia effect *Salmonella* survival in THP-1 cells.

### Host Proteomic Analysis Identifies Pathways That Support *Salmonella* Replication Within Macrophages

It had been demonstrated that EGFR pathways leads to HIF-1α activation through mTOR (32–38); therefore, we tested whether EGFR-mTOR-HIF-1α pathway is potentially used by *Salmonella* to supports its survival. To understand the role of EGFR-mTOR-HIF-1α pathway in *Salmonella* infection, we treated the infected cells with respective inhibitors like Gefitinib, Rapamycin, and Acriflavin to check whether *Salmonella* survival within the macrophages is affected. Our data indicated that the inhibition of mTOR and HIF-1α suppressed intracellular replication of S. *typhi* within the macrophages (**Supplementary Figure 2F**). Further, we wanted to understand the changes in protein profiling upon the treatment of *S. typhi*-infected macrophages with Gefitinib, Rapamycin and Acriflavin. We adapted Direct-DIA (Data independent acquisition) method to quantify changes in protein abundance of uninfected and *Salmonella* infected-THP-1 by SWATH-MS. SWATH data was median normalized (**Supplementary Figure 2B**) The quality of SWATH-MS data was assessed using the coefficient of variance (CV) and it was less than 14% over three biological replicates in all groups (**Supplementary Figure 2C**). Distribution of ration of all protein abundance was shown in **Supplementary Figure 2D**.

**Figure 2:**
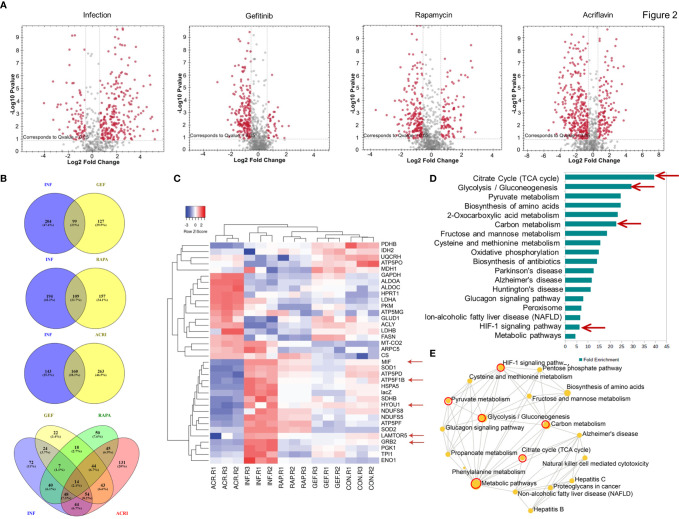
Host proteomic analysis identifies pathways that support *Salmonella* growth within macrophages. Cell lysates obtained from *S. typhi* infected and Gefitinib, Acriflavin or Rapamycin treated were used for proteomic profiling using uninfected lysates as a control. **(A)** Shows volcano plot of changes in protein abundance in infection, Gefitinib, Rapamycin and Acriflavin treatment. **(B)** Venn diagram represents a comparison of proteins between Infection and treatment groups whose p-value was <0.05 **(C)** Heat map indicating the relative expression of proteins, blue and red color indicates down and upregulation respectively same as green and red color indicates down and upregulation (n=3). **(D)** Shows significantly enriched KEGG pathway terms and their fold enrichment of 36 modulated proteins, and **(E)** Gene ontology term enrichment analysis. All 36 modulated proteins were analyzed using network analyst, related biological function is displayed in a circle, and circle size indicates the level of significance of enrichment; INF, Infection; GEF, Gefitinib; ACR, Acriflavin; RAP, Rapamycin. Data are representative of mean ± SEM from three biological replicates and one individual technical experiments.

In Direct-DIA method we build a spectral library from SWATH files that gives a library of 930 proteins and 4168 peptides. This spectral library however could be expanded using one or more combined peptide fractionation methods. This library was used for quantitative analysis of proteins in uninfected, infected, *Salmonella*-infected macrophages treated with the Gefitinib, Rapamycin and Acriflavin. Based on the statistical analysis, the changes in protein abundance was considered significant only if abundance changed > 1.5 fold with <0.05 adjusted p-value and all reported proteins and peptides were within 1% FDR (False Detection Rate). In *S. typhi*-infected macrophages, a total of 303 proteins were altered (upregulated: 225, downregulated: 78). Upon Acriflavin, Gefitinib and Rapamycin treatment of *Salmonella*-infected macrophages, 423 (Up 132, Dn 291), 226 (Up 30, Dn 196) and 266 (Up 86, Dn 180) proteins were modulated respectively (**Supplementary Table 2**). Further, we analyzed modulated proteins to find out common proteins between Infection and Gefitinib/Rapamycin/Acriflavin treatment condition; a total 99, 109, 160 proteins were commonly modulated respectively (**Figure 2B**). Volcano plot obtained from *S. typhi-*infected THP-1 cells indicate a global upregulation of 225proteins as compared to uninfected THP-1 cells most of them were reversed upon the treatment of Gefitinib, Rapamycin or Acriflavin (**Figure 2A**; **Supplementary Figure 2E**). The PCA plot indicates the distinct distribution of modulated proteins in uninfected and *S. typhi*-infected macrophages (**Supplementary Figure 2A**). More importantly, the distribution of proteins upon Gefitinib, Rapamycin or Acriflavin treatment of *S. typhi*-infected macrophages changes are significantly grouped closer to uninfected macrophages (**Supplementary Figure 2E**), indicating that Gefitinib, Rapamycin or Acriflavin treatment might be modulating the host protein to suppress *S. typhi* replication within the macrophages.

Heatmap obtained from the proteomic analysis indicated that *S. typhi-*infected macrophages causes dramatic changes in the protein abundance profile of metabolic pathway proteins like Glycolysis (TPI, PKM, Enolase, LDH, G-3-PDH), TCA cycle (Citrate Synthase, PDH, NADH DH) ATP production, Oxidative phosphorylation (G-3-PDH, LDH, NADH DH, ATP synthase, Cyt-c-Oxidase) and also importantly EGFR–mTOR–HIF1α signaling pathway was highly upregulated in infection group compared to treatment groups, as GRB2 (Growth factor receptor-binding protein 2), LTOR5 (mTOR regulating protein), HYOU 1 (Hypoxia upregulated protein 1) which are key proteins for this pathway, and these proteins are highly abundant in infection condition, whereas in treatment conditions these proteins are comparable as a control condition (**Figures 2C, D** Red arrows; **Supplementary Figure 2G–P**). Interestingly, treatment with Gefitinib, Acriflavin or Rapamycin resulted in a significant reversal of proteome changes caused by *S. typhi* infection in macrophages (**Supplementary Figure 2E**). Additionally, it was found that pathways involved in glycolysis, ATP turn over, HIF-1α signaling and TCA cycle were among others, which are majorly influenced (**Figures 2D, E** **;** **Supplementary Table 2**). Moreover, an increased level of MIF (Macrophage migration inhibition factor) in infection condition was observed, as its upregulation was known in sepsis condition (39). Interestingly, the protein abundance of MIF inhibited upon Gefitinib, Rapamycin and Acriflavin as compared to infected-macrophages (**Supplementary Figure 2P**) Taken together these data suggest that the changes observed reflect the host response to Salmonella infection.

### *Salmonella* Exploits mTOR-HIF-1α Axis to Promote Its Intracellular Survival

**Figure 3:**
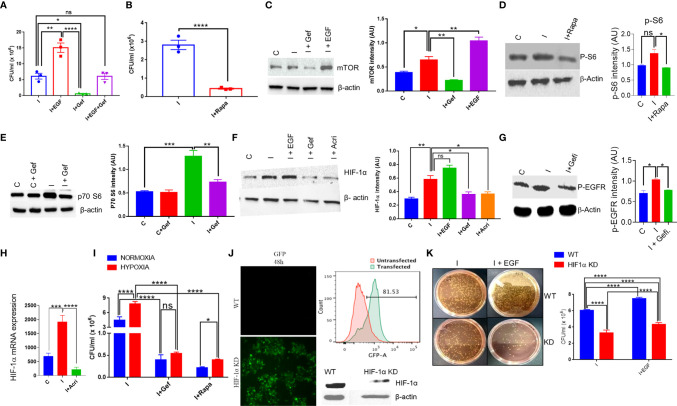
*S. typhi* exploits mTOR-HIF-1α axis to promote its own survival. **(A)** Colony-forming assay in the presence of an EGFR agonist or reverse agonist was performed for *S*. *typhi* infection in THP-1 cells. A similar assay was performed in the presence of Rapamycin, **(B)**. CFU in presence of Rapamycin. **(C–G)**. Protein expression of mTOR, p70-S6, HIF-1α and p-EGFR in THP-1 cells was determined by western blotting and quantitated by using ImageJ software in the presence or absence of infection and treatments (Panel **C-G**). **(H)** q-PCR for mRNA expression of HIF-1α in THP-1 cells with or without infection,. **(I)** Intracellular survival of *S. typhi* inside THP-1 cells under normoxia or hypoxia condition. HIF-1α guided RNA tagged with GFP was used to knock out the HIF-1α gene in THP-1 cells. Confocal microscopy images acquired 48 h after HIF-1α knockout showing high GFP expression in knockout THP-1 cells (**J** left panel), which was further confirmed by flow cytometry showing histogram overlay (**J** upper right panel); data represent two independent experiments. Western blot (**J** lower right panel) showing low expression of HIF-1α. **(K)** Intracellular survival of *S. typhi* in WT or HIF-1α knockout cells. Data are representative of mean ± SEM from three replicates and repeated three times each individual experiments *P < 0.05, **P < 0.01, ***P < 0.001, ****P < 0.0001 (Student’s t-test and one way ANOVA).

To understand the intracellular pathway of the macrophages, which is exploited by *S. typhi* for its replication within the macrophages, downstream signaling of the EGFR pathway was investigated. One of the downstream signaling of EGFR involves mTOR and HIF-1α axis, which was also found to be significantly affected by *Salmonella* infection in THP-1 cells (40). We first tested whether EGF, one of the ligands of EGFR, can promote the intracellular replication of S. *typhi* within the macrophages. Our data indicated that EGF enhanced the intracellular survival of S. *typhi* within the macrophages **(** **Figure 3A** **)**. Interestingly, Gefitinib treatment suppressed the EGF-mediated intracellular replication of S. *typhi* within the macrophages **(** **Figure 3A** **)**, indicating that EGFR activity is critical for the intracellular survival of *S. typhi*.

Since mTOR is one of the major signaling targets downstream of EGFR, therefore we tested whether blocking of mTOR activity by Rapamycin, a known antagonist for mTOR, could affect the intracellular survival of S. *typhi* within the macrophages. It was found that inhibiting mTOR activity by Rapamycin results in about 90% inhibition of intracellular replication of *S. typhi* as indicated by CFU count **(** **Figure 3B** **)**, indicating a potential role of mTOR in intracellular replication of *S. typhi*. We measured the changes in protein level expression of molecules involved in mTOR signaling by western blot analysis. As indicated, S. *typhi* infected macrophages increases both mTOR and p70-S6 kinase levels (**Figures 3C, E**). Interestingly, EGF treatment further enhanced the mTOR expression in S. *typhi* infected macrophages (**Figure 3C**), indicating that EGFR signaling enhances the mTOR. Moreover, Gefitinib treatment found to decrease both mTOR and p70-S6 kinase (**Figures 3C, E**). We also observed that both Gefitinib and Rapamycin treatment inhibits phosphorylation of EGFR and p-S6 (Activation state of mTOR) respectively (**Figures 3D, G**). To further understand whether *S. typhi* infection in THP-1 increases HIF-1α at protein level. Our Western blotting data clearly indicated that *S. typhi*-infected THP-1 macrophages enhanced HIF-1α protein as compared to uninfected THP-1 macrophages (**Figure 3F**). Interestingly, treatment of *S. typhi*-infected macrophages with EGF further increased the HIF-1α while Gefitinib and Rapamycin treatment of *S. typhi*-infected THP-1 macrophages suppressed HIF-1α expression, indicated that *S. typhi* -induced EGFR and mTOR signals converge to HIF-1α. Moreover, our data indicates that *S. typhi* also induced the mRNA expression of HIF-1α in infected THP-1 macrophages, which is suppressed by Acriflavin (**Figure 3H**). Since HIF-1α s induced during hypoxic conditions, therefore we further studied whether hypoxic condition supports the intracellular replication of *S. typhi* within the macrophages. Consistently, culturing *S*. *typhi*- infected THP-1 macrophages under experimental hypoxic conditions was found to facilitate intracellular replication of bacteria as compared to normoxic conditions (**Figure 3I**). Interestingly, EGFR and mTOR inhibition through Gefitinib and Rapamycin respectively, significantly suppressed the intracellular replication of *S. typhi* within macrophages **(** **Figure 3I** **)**, further supporting that EGFR-mTOR activation supports intracellular replication of *S. typhi* within THP-1 macrophages by enhancing HIF-1α.

We further confirmed the role of HIF-1α in supporting the intracellular replication of *S. typhi* within the macrophages by knock-out HIF-1α using CRISPR-Cas9 in THP-1 cells. CRISPR-Cas9-mediated knock-out of HIF-1α in THP-1 cells was confirmed by GFP expression **(** **Figure 3J** **)**, which was further confirmed by flow cytometry and HIF-1α protein expression (**Figure 3J** right upper & below panel respectively). The intracellular survival of *S. Typhi* was then studied in WT or HIF-1α-KO non clonal THP-1 cells through colony-forming assay. HIF-1α-KO resulted in a significant loss of ability for intracellular survival of *S. typhi* (**Figure 3K**), corroborating our previous observations using pharmacological inhibition of HIF1α. Taken together, these data indicated the EGFR-mTOR-HIF-1α axis is essential for the intracellular replication of *Salmonella*.

### Inhibition of EGFR Signaling Suppresses *S. Typhimurium* Infection in Mice

**Figure 4:**
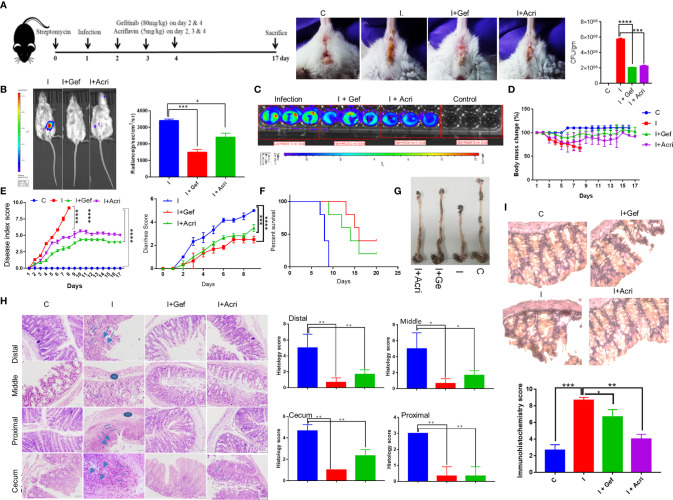
Inhibition of EGFR signaling suppresses S. Typhimurium infection in mice. Recovery of chronically S. Typhimurium infected FVB mice was studied in the presence of Gefitinib or Acriflavine. **(A)** Shows the pictorial representation of the dose regime for the establishment of infection and Gefitinib/Acriflavin treatment. Panel a (right), shows mouse images indicating diarrhea index for 6^th^ post-infection. The stool was collected and plated on SS agar to determine S. Typhimurium colony formation (a, Far right). **(B)** In vivo imaging and intensity quantitation (right panel) of FVB mice infected with S. Typhimurium strain (Xen33) expressing luciferase on the 6^th^ day (n=5 mice per group). **(C)** Luminescence imaging of stool obtained from Xen33 infected mice, data indicates three independent experiments. Panel **(D–F)** shows the percentage change in body mass, disease index score, diarrhea score and percent survival (Kaplan Meier survival analysis) for FVB mice infected with S. Typhimurium in the presence or absence of Gefitinib or Acriflavin (n=5 mice per group). **(G)** The image shows the length of the colon isolated from treated or untreated mice. **(H)** H & E staining (10X) of the proximal, middle and distal colon along with cecum showing regions of ulcers (arrow), inflammation (line), edema(circle)of different groups along with their histological scores. **(I)** Immunohistochemistry images (40X) showing expression of HIF-1α and its quantification in the colon (n=5). Data representative of triplicates and repeated three times each individual experiments *P < 0.05, **P < 0.01, ***P < 0.001, ****P < 0.0001, ns-non-significant. (Student’s t-test and one-way ANOVA).

To study the *in vivo* relevance of our findings, we studied S. Typhimurium infection in FVB mice, which is known as acute enteritis and chronic animal model to investigate the therapeutic potential of Gefitinib treatment (1, 4, 41). *S*. Typhimurium infected mice treated with Gefitinib or Acriflavin as indicated in (**Figure 4A**, left panel) showed lower diarrhea associated with lower S. Typhimurium burden as compared to infection (**Figure 4A**, middle and right panel). We further use *in vivo* imaging to confirm our findings using genetically engineered luciferase-expressing Xen33 (FDA 1189) *Salmonella* infected FVB mice. Our data showed the lower intensity of luciferase signal for mice treated with Gefitinib or Acriflavin at day 5th (**Figure 4B**, left panel). The luciferase signal was further quantitated, which showed a 2.3 fold lower bacterial burden in the gut in Gefitinib treated mice as compared to the control group (**Figure 4B**, right panel). The bioluminescence analysis in the stool of Xen33 infected mice groups also showed a significantly lower number of bacteria in Gefitinib or Acriflavin treated group as displayed by decreased luminescence intensity (**Figure 4C**). Besides, the other infection parameters such as percent change in body mass, diarrhea, disease index, and animal survival showed significant recovery in Gefitinib and Acriflavin treated mice as compared to S. Typhimurium infected groups (**Figures 4D–F**). Moreover, the food and water intake of Gefitinib and Acriflavin group were found to be similar to that of healthy control (**Supplementary Figure 3**). It was found S. Typhimurium infection in mice significantly reduced the colon length as compared to healthy control mice (**Figure 4G** **),** however Gefitinib or Acriflavin treatment of S. Typhimurium infected mice restored the length of the colon as compared to the infection group (**Figure 4G**). It is well established that within the gut S. Typhimurium infects and colonizes in the colon where it causes severe inflammation, associated with neutrophils and macrophages infiltration and edema. Histological examination of colon and cecum using H & E staining for the section of the proximal, middle and distal colon, as well as cecum showed that Gefitinib and Acriflavin treatment leads to a significant recovery in the gross anatomy of colon and cecum as compared to the chronically infected FVB mice groups (**Figure 4H**). Further, immunohistochemistry for HIF-1α expression was carried out in the colon sections to investigate the role of HIF-1α in disease progression. Our results indicate that infection of S. Typhimurium leads to increased HIF-1α expression in the colon, which was significantly reduced upon Gefitinib and Acriflavin treatment (**Figure 4I**). Overall, these *in vivo* observations ascertained that EGFR-HIF1α is essential to establish infection *in vivo*.

### Blocking EGFR Signaling Reprograms Metabolomic Changes in *S. Typhimurium* Infection

**Figure 5:**
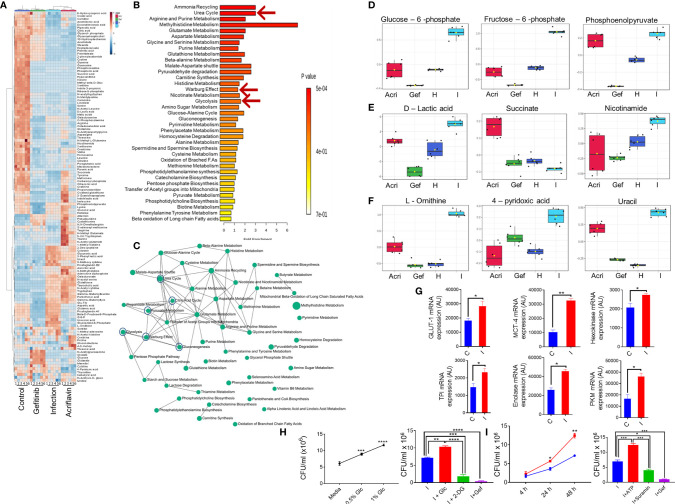
Blocking EGFR signaling reprograms metabolomic changes in *S.* Typhimurium infection. Serum obtained from S. Typhimurium infected FVB mice receiving Gefitinib or Acriflavin treatment was used for metabolomics profiling using healthy serum control. **(A)** Heat map indicating changes in the metabolites for different groups (n=6 mice per group). **(B, C)** Pathway enrichment and pathway network. **(D-F)** shows the distribution of important metabolites for glycolysis, Warburg effect and urea cycle across different groups. **(G)** mRNA expression of genes involved in glucose transport and glycolysis in the presence or absence of S. Typhimurium infection in BMDM cells, bars show duplicate values for each gene. **(H, I)** dose kinetics for glucose and ATP response against intracellular replication of S. Typhimurium inside BMDM cells, Data are representative of mean ± SEM from one individual experiments *P < 0.05, **P < 0.01, ***P < 0.001, ****P < 0.0001 (Student’s t-test).

In order to understand the mechanism underlying host-directed immunity against *Salmonella* infection by Gefitinib, we carried out serum metabolomic profiling for S. Typhimurium infected mice with or without Gefitinib or Acriflavin treatment and compared their metabolomic profile with the healthy FVB mice. Our results obtained on MetaboAnalyst software shows that all the four groups (healthy, infected, infected plus Gefitinib and infected plus Acriflavin) showed less variation within the group as revealed by 3D PCA and PLSDA plots (**Supplementary Figure 4A** left and right respectively). Further, the abundance of individual metabolites across four groups was converted on the intensity scale of 3 to -3 and plotted as a heatmap. The results showed global metabolic changes during chronic S. Typhimurium infection in FVB mice. As shown in the heatmap, S. Typhimurium infection changes metabolites as compared to healthy group (**Figure 5A**). Interestingly, these changes in the metabolomic profile were found to be partly restored from disease profile by the treatment of Gefitinib and to a lesser extent by Acriflavin (**Figure 5A**). Remarkably, the metabolomic pathways which were significantly affected by S. Typhimurium infection and then restored by Gefitinib treatment were found to be those involving the Warburg effect, glycolysis, ATP turnover, Urea, etc. (**Figures 5B, C**; **Supplementary Figure 4C-L**). Interestingly, metabolites involved in glycolysis such as Glucose-6-phosphate, Fructose-6-phosphate and phosphoenolpyruvate (**Figure 5D**), Warburg effect and ATP turn-over such as D-lactic acid, succinate, Nicotinamide (**Figure 5E**) and Urea cycle such as L-ornithine, 4-pyridoxic acid and uracil (**Figure 5F**) were found to be significantly perturbed upon Gefitinib treatment indicating a global metabolomic reprogramming. Furthermore, enzymes and transporters involved in glucose influx and glycolysis were also found to be significantly upregulated under *Salmonella* infection (**Figure 5G** **;** **Supplementary Figure 4B–L**). To validate the role of glucose and ATP, as suggested in the metabolomic profile, promoting the intracellular replication of *S. typhi* in macrophages. Interestingly, *S. typhi*-infected THP-1 macrophages cultured with increasing concentration of glucose showed an increase in intracellular replication of *S. typhi* **(** **Figure 5H**, left panel), suggesting that increased glucose concentration might be enhancing the rate of glycolysis which might in turn be promoting the replication of *Salmonella* within the macrophages. To support this, we cultured *S. typhi*-infected THP-1 macrophages with 2-DG, an inhibitor of glycolysis, and found that 2-DG treatment was able to suppress the intracellular replication of *S. typhi* within the macrophages **(** **Figure 5H**, right panel). We further validated the role of host ATP, as suggested by metabolic profile, in the intracellular replication of *Salmonella*. Increasing concentration of ATP enhanced the *S. typhi* replication within the macrophages **(** **Figure 5I**, left panel). In contrast, treatment of *S. typhi*-infected macrophages with Suramin, a blocker of ATP, suppressed the intracellular replication of S. *typhi* **(** **Figure 5I**, right panel). Interestingly, Gefitinib was able to limit the ATP generation from the host cells (**Supplementary Figure 5B**). All these results suggested that both glucose metabolism and ATP generation affected by the Gefitinib and inhibit the replication of *Salmonella* inside the host.

### EGFR-HIF1α Axis Is Essential to Promote MDSCs in *Salmonella* Infection

**Figure 6:**
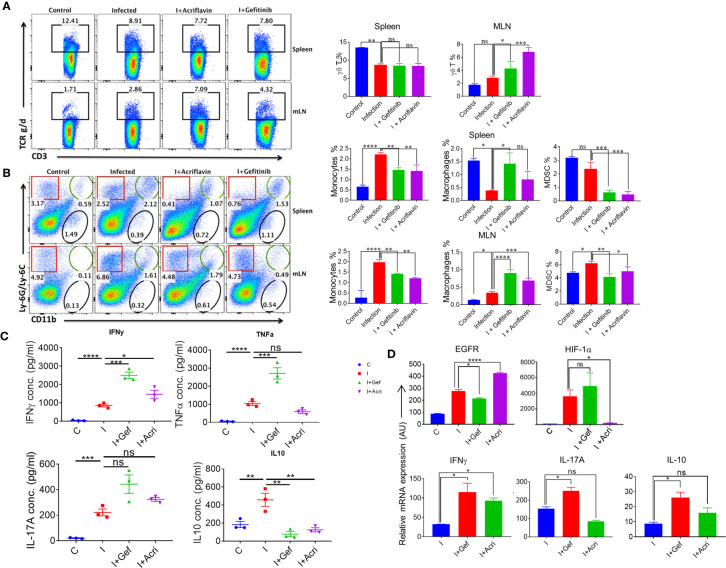
EGFR-HIF1α axis is essential to promote MDSCs in *Salmonella* infection. **(A, B)** Dot plots and bar graphs indicating % frequency of γδ T cells, macrophages, MDSCs, monocytes in spleen and MLN in mice infected with S. Typhimurium (n=3 mice per group), analyzed using Flowjo. **(C)** Graph showing the concentration of different pro and anti-inflammatory cytokine (IFN-γ, TNF-α, IL-17a, IL-10) in the blood serum (n=3), data representative of triplicates *P < 0.05, **P < 0.01, ***P < 0.001, ****P < 0.0001 (one way ANOVA). **(D)** Bar graph for mRNA expression of EGFR, HIF1α, IFNγ, IL17A, and IL10 in the MLN cells, data representative of triplicates *P < 0.05, **P < 0.01, ***P < 0.001, ****P < 0.0001 a-d. one way ANOVA.

To understand the immunological response mediated by EGFR and HIF-1α during *Salmonella* infection, we infected the mice with S. Typhimurium and treated them with Gefitinib or Acriflavin. As shown in Figure 4, both Gefitinib and Acriflavin suppressed S. Typhimurium in mice; the immune population in lymphoid organs (mesenteric lymph nodes and spleen) was characterized in S. Typhimurium*-*infected FVB mice. Our results demonstrated that there was a significant increase in γδ T cells in MLN upon the treatment with Gefitinib and Acriflavin in infected mice (**Figure 6A**, right and left panel). We further tested monocytes, macrophages and myeloid-derived suppressor cells (MDSCs) population in both spleen and MLN in these animals. While monocytes frequency is increased upon *S*. Typhimurium infection in both spleen and MLN, Gefitinib and Acriflavin treatment suppressed the frequency of monocytes in S. Typhimurium infected mice. Moreover, myeloid-derived suppressor cells (MDSCs) population was found to be significantly reduced by Gefitinib and Acriflavin treatment in S. Typhimurium infection (**Figure 6B**), indicating a possible role of MDSCs in driving the *Salmonella* infection *in vivo*.

To understand the status of overall immune activation upon the treatment of S. Typhimurium-infected mice with either Gefitinib or Acriflavin, serum circulating cytokines levels were measured by ELISA. Interestingly, the level of IFN-γ, TNF-a, IL-17A which is the major cytokine required for mounting potent immunity against intracellular pathogen, was found to upregulate (**Figure 6C**). Similar changes were also observed in IL-1β levels during infected THP-1 cells (**Supplementary Figure 5C**). Level of IL-10, an anti-inflammatory cytokine, was found to be downregulated upon Gefitinib or Acriflavin treatment as compared to S. Typhimurium*-*infection (**Figure 6C**). mRNA expression for EGFR, HIF-1α, IFNγ, IL-17A and IL-10 genes from MLN isolated from S. Typhimurium infected FVB mice in the presence or absence of Gefitinib or Acriflavin showed similar expression profiles (**Figure 6D**). These observations together demonstrated that both Gefitinib and Acriflavin suppress the replication of *Salmonella* by enhancing pro-inflammatory response while suppressing the MDSCs.

## Discussion

*Salmonella* infection is one of the leading causes of enteric fever in humans, arising due to gastroenteritis with an estimated 11-20 million new cases with about 128000-161000 cases of mortality annually (1, 2, 42). To treat the drug resistant strains of *Salmonella*, an alternative strategies are required to combat the life-threatening infections of multi-drug resistant *Salmonella*. Three such exciting alternatives include vaccine development, phage therapy, and use of compounds for the host-directed immunity (10–12, 43). Among these, HDT provides a rapid yet cost-effective strategy to counter the growing challenge posed by drug-resistant bacteria. In the current study, we screened an FDA approved library of 61 compounds known to target distinct host functions, such as cholesterol distribution on the plasma membrane, G-protein coupled receptors and intracellular calcium signaling, for their potential host-mediated therapeutic effects against *Salmonella* infection in macrophages. Our analysis of intracellular survival inhibition during the establishment of *S. typhi* infection followed by post-exposure of the compounds for 24h showed various degrees of inhibitions for all 61 compounds. Additionally, inhibition of internalization based on pre-exposure of the compound followed by *S. typhi* infection, suggesting that most of the compounds hold the minimal inhibitory potential for internalization of *S*. *typhi*. Among all compounds, we found Gefitinib significantly suppressed both intracellular and uptake of *Salmonella*, indicating the possibility of *Salmonella* modulating host EGFR pathway for its own survival as Gefitinib targets EGFR signaling (10, 26, 44–46). Dirk Brehmer et al., in 2005 has described the 20 new cellular targets of the Gefitinib besides EGFR that might behave as off-targets (47). But we haven’t looked at other cellular targets as EGFR is the most prominent and well-studied target for Gefitinib. Interestingly, *Salmonella* exploit EGFR for its entry and subsequently internalization through flagellin in gut of the host (48–52). Since Gefitinib blocks EGFR phosphorylation, it is possible that *Salmonella* entry and uptake by host cells require EGFR activation (**Figure 3G**) (31, 48, 49, 53–55). Further downstream of the EGFR, one of the main important factor for survival of intra cellular bacteria, is mTOR. The role of Rapamycin in inhibiting the mTOR phosphorylation had been described previously by Ballou and Lin (56), and Thoreen et al. (57) (56, 58). We have also reported that Rapamycin inhibits phosphorylation of p-S6, which is the activation state of m-TOR and upregulated in presence of infection (**Figure 3D**). Consistently EGF, as indicated by our data, promotes intracellular replication of *Salmonella* within the macrophages. It might be possible that flagellin, together with EGF synergistically activate EGFR signaling to promote the uptake of *Salmonella*. Moreover, Gefitinib which blocks EGFR, for uptake of bacteria (10, 59). In addition, our proteomics analysis suggests the involvement of HIF-1α, further suggesting a possibility that EGFR-HIF-1α axis in intracellular uptake and survival of the *Salmonella* within the host. As Gefitinib and Rapamycin are known pharmacological inhibitors of EGFR and mTOR respectively, we hypothesized that this pathway could be modulated or beneficial to the *Salmonella*. This hypothesis is also supported by our *in vitro* experiments (western blot, CRISPR knock out experiment, RT-PCR).

Previous reports demonstrated that blocking of EGFR signaling suppresses mTOR pathway. In addition, Rapamycin treatment affects EGF/EGFR signaling and causes diverse physiological changes within the cell (48–50, 60). Several studies indicated that mTOR play a pivotal role in the replication of intracellular pathogens (37, 53). Our data suggests that mTOR is critical for *S*. *typhi* survival within the macrophages. Consistently, expression of both mTOR and p70-S6 kinase was significantly upregulated upon infection with *Salmonella*. It is well known that mTOR pathway play an important role in intra cellular pathogens survival (61). Interestingly, Gefitinib may also act by inducing autophagy in host cells as indicated by our LC3B expression study (**Supplementary Figure 5A**).

Our data indicates the EGFR-mTOR ultimately activate HIF-1a to further support the replication of the *Salmonella*. HIF-1α is essential transcription factor that induces hypoxia within the cells. Hypoxia or low oxygen concentration is an environmental stimulus that induces several physiological changes within the cells (62). Upon infection, oxygen can be destitute from tissues due to vascular damage or intensive metabolic activity induced by pathogen, which leads to a hypoxic environment within the cells (16). Hypoxia stabilizes HIF-1α to further promote hypoxia-induced survival of the bacteria. Acriflavin and Rapamycin has been shown to block the survival of intracellular pathogen such as *Staphylococcus aureus*, *Escherichia coli*, *Streptococcus agalactiae*, *Pseudomonas aeruginosa*, *Bartonella henselae*, *Yersinia enterocolitica*, and *Chlamydophila pneumoniae* (11, 21, 49, 62–65). Our results indicated the enhanced expression of HIF-1α during *S. typhi* infection at both mRNA and protein levels. Inhibition of EGFR using Gefitinib suppressed mTOR and HIF-1α as well as inhibited intracellular survival of *S. typhi* indicates that *S. typhi* utilizes mTOR-HIF-1α axis for its replication. Consistently, our data suggested that the intracellular survival of *S. typhi* was significantly reduced in HIF-1α-KO as compared to the WT macrophages (**Figure 3I**).

Our *in vitro* data indicated that EGFR pathway is crucial for the establishment of *Salmonella* infection. Consistently, Gefitinib treatment of S. Typhimurium*-*infected mice showed recovery associated with inhibition of HIF-1α, further emphasizing the critical role EGFR-HIF-1α axis regulating *Salmonella* infection *in vivo*. Gefitinib treatment induced proteomic and metabolomic reprogramming of *Salmonella* infected macrophages, indicating that *Salmonella* utilizes EGFR signaling to modulate host protein and metabolites to establish infection. Interestingly, our data demonstrated that *S. typhi* infection of THP-1 cells upregulated IL-1β secretion thereby directly regulating epithelial lining of the gut. MLN is one of the important secondary lymphoid organs in the context of the gut immunity that develop a successful immunological response against the pathogens that invade through oral route (66–68). Our immunophenotyping data suggested that Gefitinib treatment increases macrophage frequency while suppressing myeloid-derived suppressor cells (MDSCs) in both MLN and spleen, which corroborates with previous reports (69–72). These data further suggest a possibility that *Salmonella* enhance the frequency of suppressor MDSCs population. While Gefitinib treatment in *Salmonella*-infected mice enhanced pro-inflammatory cytokines like IFN-γ, TNF-α, IL-17, it suppressed IL-10 secretion indicating that inhibition of EGFR signaling triggers a strong inflammatory response in the gut, which is essential for successful immunity against *Salmonella*.

Our *in vitro* and *in vivo* data strongly suggests that HIF-1α plays a crucial role in regulating *Salmonella* infection. HIF-1α is an important transcription factor that is known to influence the metabolomic status of the cell along with tissue inflammation (73, 74). Our results suggested that Gefitinib, Rapamycin and Acriflavin induces global proteomic changes and also metabolomics reprogramming within the host by perturbing the balance of ATP turn over, glycolysis and Warburg effect. It has been previously reported that *Salmonella* infection creates Warburg like effect within the host cell thereby shifting the axis toward glycolysis. Interestingly, we found Gefitinib induced reprogramming of metabolism to be closely overlapping the proteo-metabolomics profile of the healthy, demonstrating further that Gefitinib mediated reprogramming is crucial for its host-directed antimicrobial activity. Using the compound library together with proteomics and metabolomics, we identified factors/pathways that are modulated by EGFR-HIF-1α pathways in *Salmonella* infection, and therefore hypothesize that blocking these factors/pathway during *Salmonella* infection may lead to provide new targets of HDT for eliminating intracellular pathogens.

## Data Availability Statement

The mass spectrometry proteomic data presented in the study are publicly available. This data can be found here: http://www.ebi.ac.uk/pride/archive/projects/PXD024771.

## Ethics Statement

The animal study was reviewed and approved by the institutional animal ethics committee—THSTI.

## Author Contributions

AA has conceptualized, wrote the paper and supervised the study. SS designed, performed the experiments, collected, analyzed the data, and wrote the paper. RG: performed the proteomics and YK, SS, ZR has performed the serum metabolomics experiment and analysis. BK, TM: Proteomics analysis. AP has provided the Enzo library, MP has performed the experiments, ZR, RP performed the experiments analysed the data. DR, SS: FACS analysis. BK Bioinformatic analysis and RD helped in imaging and analysis. AJ: Advised in performed the experiments, critical inputs in the editing of the paper. All authors contributed to the article and approved the submitted version.

## Funding

This work was supported by a core grant of the Translational Health Science and Technology Institute (THSTI/T001). SS supported by the Indian Council of Medical Research (ICMR) SRF fellowship (80/1004/2016-ECD-1).

## Conflict of Interest

The authors declare that the research was conducted in the absence of any commercial or financial relationships that could be construed as a potential conflict of interest.

## Supplementary Material

Supplementary Figure 1 Identification of Gefitinib as a potent drug against *S. typhi.* **(A)** Cytotoxicity assay of compounds. Left panel shows there was no cytotoxicity or direct killing of *Salmonella*, the right panel shows no cytotoxicity on THP-1 cells. **(B)** The bar graph represents % inhibition ± SEM of intracellular replication of *S. typhi* after the post-exposure of Enzo library compounds (1µM). **(C)** The number of compounds showing <50%, >50%, >80%, >90% inhibition of intracellular survival on dose dependent kinetics (1µM, 100nM, 10nM). **(D)** The number of compounds showing invasion inhibition in THP-1 cells from below 50% to above 50%, >80%, >90%. **(E, F)** Percentage Intra-cellular and invasion inhibition of 3 compounds (dose kinetics assay) in caco2 cells. Data represents mean ± SEM from three replicates and repeated three times each individual experiments.

Supplementary Figure 2 Relative abundance of proteins upon *S. typhi*-infected macrophages and treatment with Gefitinib, Acriflavin or Rapamycin. **(A)** Depicts PCA distribution for individual groups. **(B)** For quantitative analysis, Total intensity count (TIC) is used for normalization (median) among groups. **(C)** Density plot shows the precursor % CV distribution for all groups. **(D)** Histogram of average log2 ratio shows the data distribution of all proteins. **(E)** Heat map indicating the relative expression of proteins in different groups. **(F)** Intra cellular survival inhibition of *Salmonella* was observed in THP-1 cells, in the presence of Rapamycin and Acriflavin. **(G–P)** Demonstrate different key protein modulations in infection and treatment conditions which are important in Glycolysis, TCA cycle, ATP synthesis, Carbon metabolism. **(N)** mTOR regulatory protein (LAMTOR5) highly upregulated in infection conditions. **(O)** Hypoxia-inducible factor -1α upregulated protein (HYOU1) was upregulated in infection whereas in treatment it was comparable as control. ****P < 0.0001 (One way ANOVA) **(P)** Macrophage inhibition factor (MIF) downregulated in treatment conditions. Data representative of triplicates and one technical experiments *P < 0.05, **P < 0.01, ***P < 0.001, ****P < 0.0001 (one way ANOVA).

Supplementary Figure 3 In vivo effect of treatment on S. Typhimurium infection and disease progression in FVB mice. **(A, B)** Food and water uptake in different groups. Left panel a Graph show that food and panel b shows water uptake indicates an almost similar pattern in treatment groups as compared to the infection group (n=5 mice per group). Data are representative of mean ± SEM from five biological replicates.

Supplementary Figure 4 Metabolomic reprogramming of metabolites from *S.* Typhimurium infected and treated serum samples. **(A)** Shows 3D distribution on PCA (left) and PLSDA (right) plot for individual groups (n=6). **(B)** Overview of pathways, (Dark Color circles shows as highly upregulated pathway, light Color circles shows less compare to dark circle) **(C–L)** Shows that modulation of different metabolites of Glycolysis, TCA cycle, Urea cycle, Gluconeogenesis pathways (n=6). Data represents six biological replicates and one technical experiment.

Supplementary Figure 5 Gefitinib, Acriflavin decreases ATP synthesis and IL-1β production in BMDMs respectively. **(A)** Gefitinib plays an important role in autophagy. **(B)** *Salmonella* induces ATP synthesis in macrophages, which was drastically decreased in Gefitinib, Acriflavin, and Suramin treated conditions respectively. **(C)** *S. typhi* infection of THP-1 cells augments IL-1β secretion, data representative of triplicates ***P < 0.001, ****P < 0.0001 (one way ANOVA).

Supplementary Table 1 Enzo compound library used for screening for intracellular survival and invasion inhibition of *Salmonella* in THP-1.

Supplementary Table 2 Number of protein hits identified through proteomics based on the cut of > 1.5 fold with <0.05 p-value and subset of Proteins identified having distinct metabolic pathways, Shortlisted proteins with differential expression after normalization with the control sample.
