# Supplementary figures and images for: Gefitinib Results in Robust Host-Directed Immunity Against Salmonella Infection Through Proteo-Metabolomic Reprogramming
Source: Front Immunol. 2021 Mar 31;12:648710. doi: 10.3389/fimmu.2021.648710 (PMC8044459; doi:10.3389/fimmu.2021.648710)

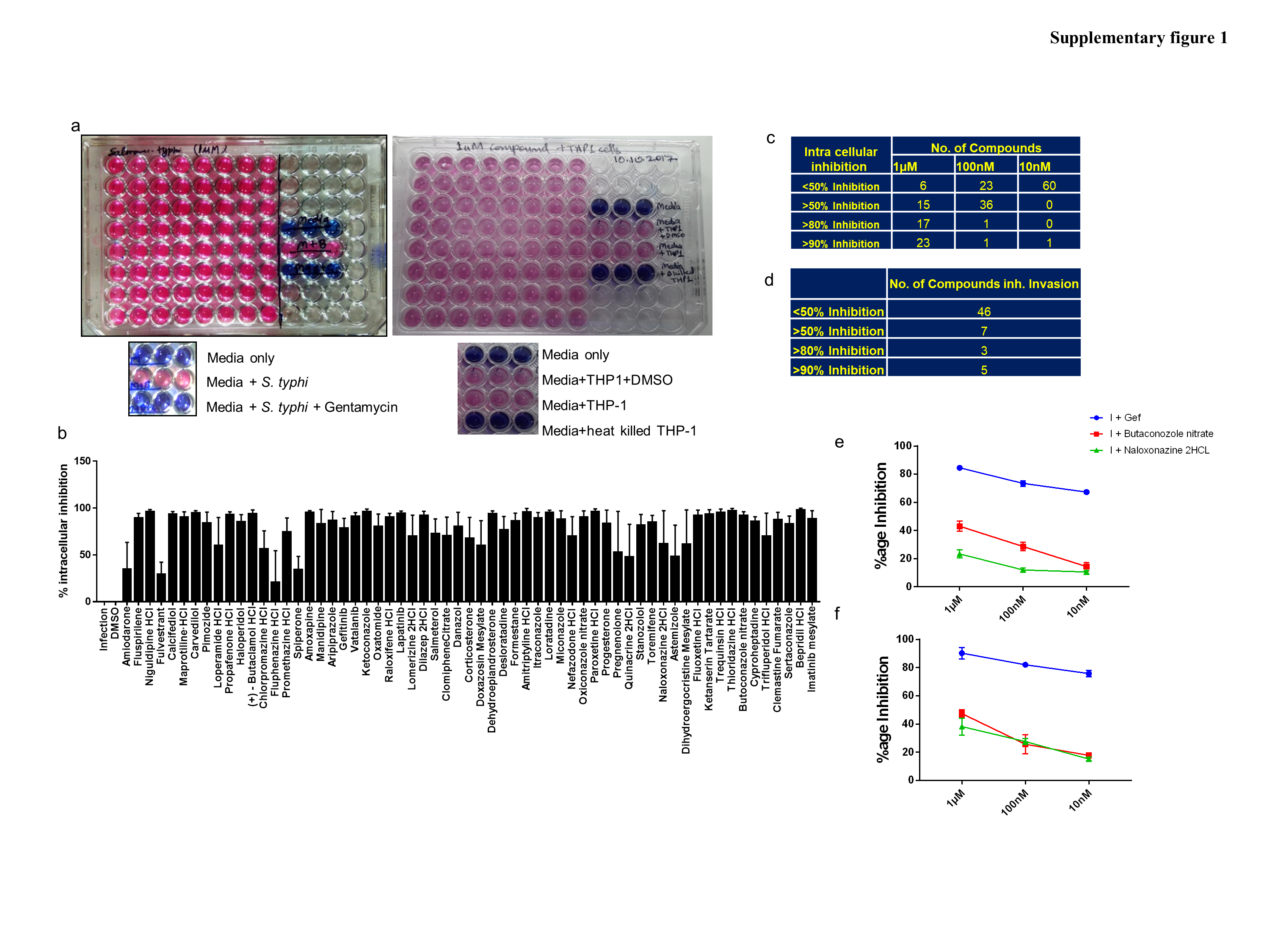

Supplement: Supplementary Figure 1 — Identification of Gefitinib as a potent drug against S. typhi. (A) Cytotoxicity assay of compounds. Left panel shows there was no cytotoxicity or direct killing of Salmonella, the right panel shows no cytotoxicity on THP-1 cells. (B) The bar graph represents % inhibition ± SEM of intracellular replication of S. typhi after the post-exposure of Enzo library compounds (1µM). (C) The number of compounds showing <50%, >50%, >80%, >90% inhibition of intracellular survival on dose dependent kinetics (1µM, 100nM, 10nM). (D) The number of compounds showing invasion inhibition in THP-1 cells from below 50% to above 50%, >80%, >90%. (E, F) Percentage Intra-cellular and invasion inhibition of 3 compounds (dose kinetics assay) in caco2 cells. Data represents mean ± SEM from three replicates and repeated three times each individual experiments. [file Presentation_1.zip › Supplementary 1.TIF]

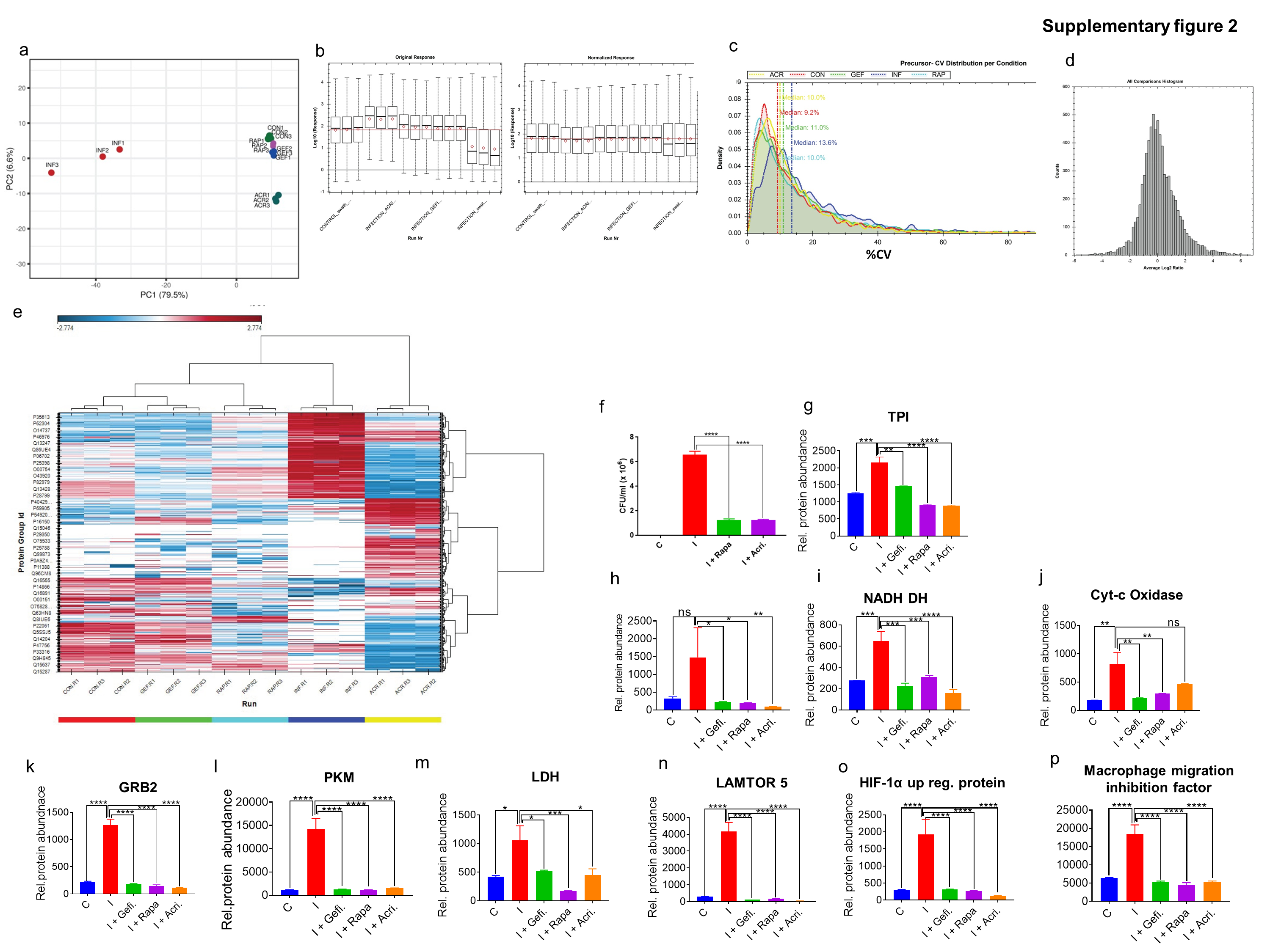

Supplement: Supplementary Figure 1 — Identification of Gefitinib as a potent drug against S. typhi. (A) Cytotoxicity assay of compounds. Left panel shows there was no cytotoxicity or direct killing of Salmonella, the right panel shows no cytotoxicity on THP-1 cells. (B) The bar graph represents % inhibition ± SEM of intracellular replication of S. typhi after the post-exposure of Enzo library compounds (1µM). (C) The number of compounds showing <50%, >50%, >80%, >90% inhibition of intracellular survival on dose dependent kinetics (1µM, 100nM, 10nM). (D) The number of compounds showing invasion inhibition in THP-1 cells from below 50% to above 50%, >80%, >90%. (E, F) Percentage Intra-cellular and invasion inhibition of 3 compounds (dose kinetics assay) in caco2 cells. Data represents mean ± SEM from three replicates and repeated three times each individual experiments. [file Presentation_1.zip › Supplementary 2.tif]

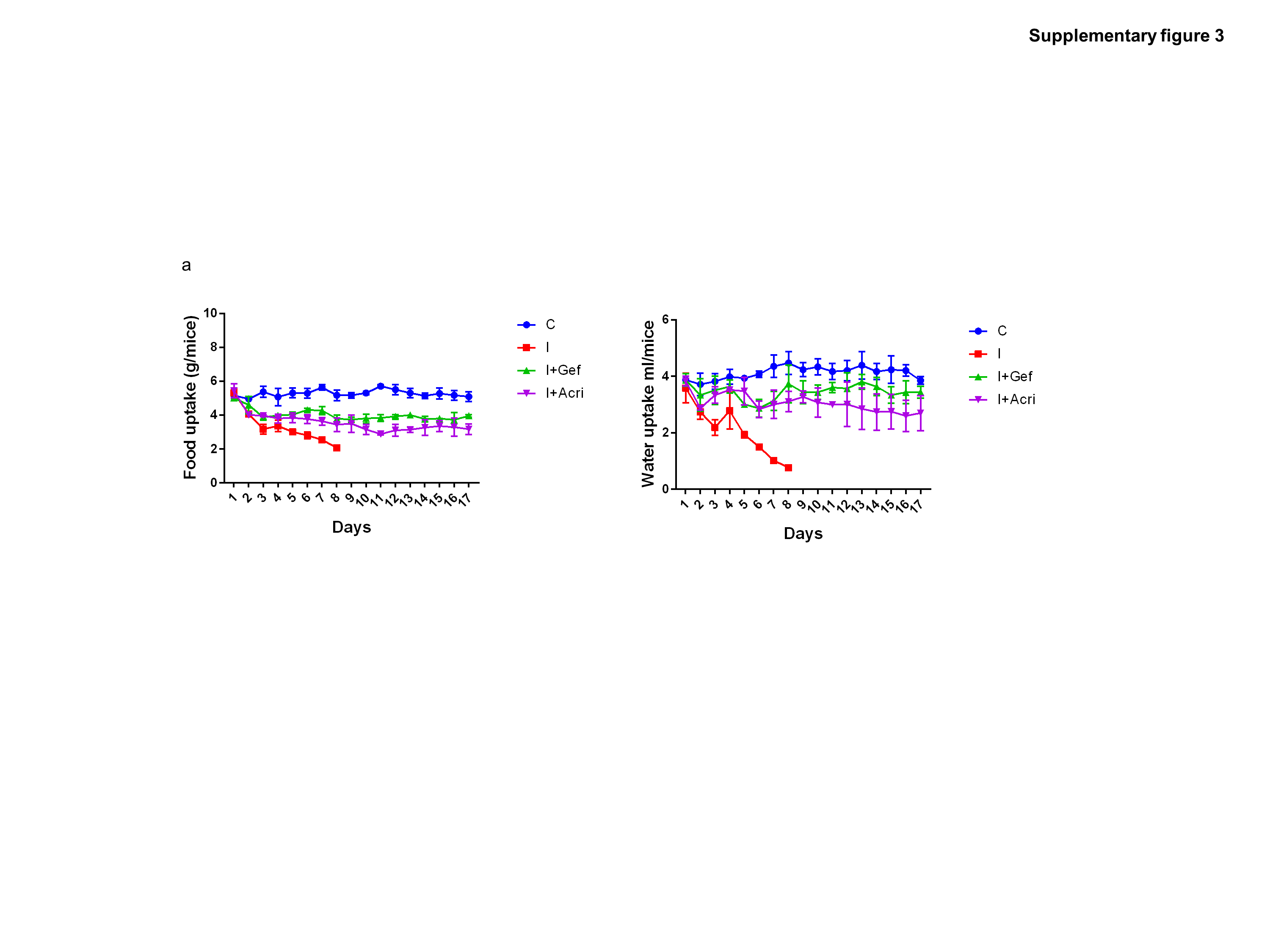

Supplement: Supplementary Figure 1 — Identification of Gefitinib as a potent drug against S. typhi. (A) Cytotoxicity assay of compounds. Left panel shows there was no cytotoxicity or direct killing of Salmonella, the right panel shows no cytotoxicity on THP-1 cells. (B) The bar graph represents % inhibition ± SEM of intracellular replication of S. typhi after the post-exposure of Enzo library compounds (1µM). (C) The number of compounds showing <50%, >50%, >80%, >90% inhibition of intracellular survival on dose dependent kinetics (1µM, 100nM, 10nM). (D) The number of compounds showing invasion inhibition in THP-1 cells from below 50% to above 50%, >80%, >90%. (E, F) Percentage Intra-cellular and invasion inhibition of 3 compounds (dose kinetics assay) in caco2 cells. Data represents mean ± SEM from three replicates and repeated three times each individual experiments. [file Presentation_1.zip › Supplementary 3.TIF]

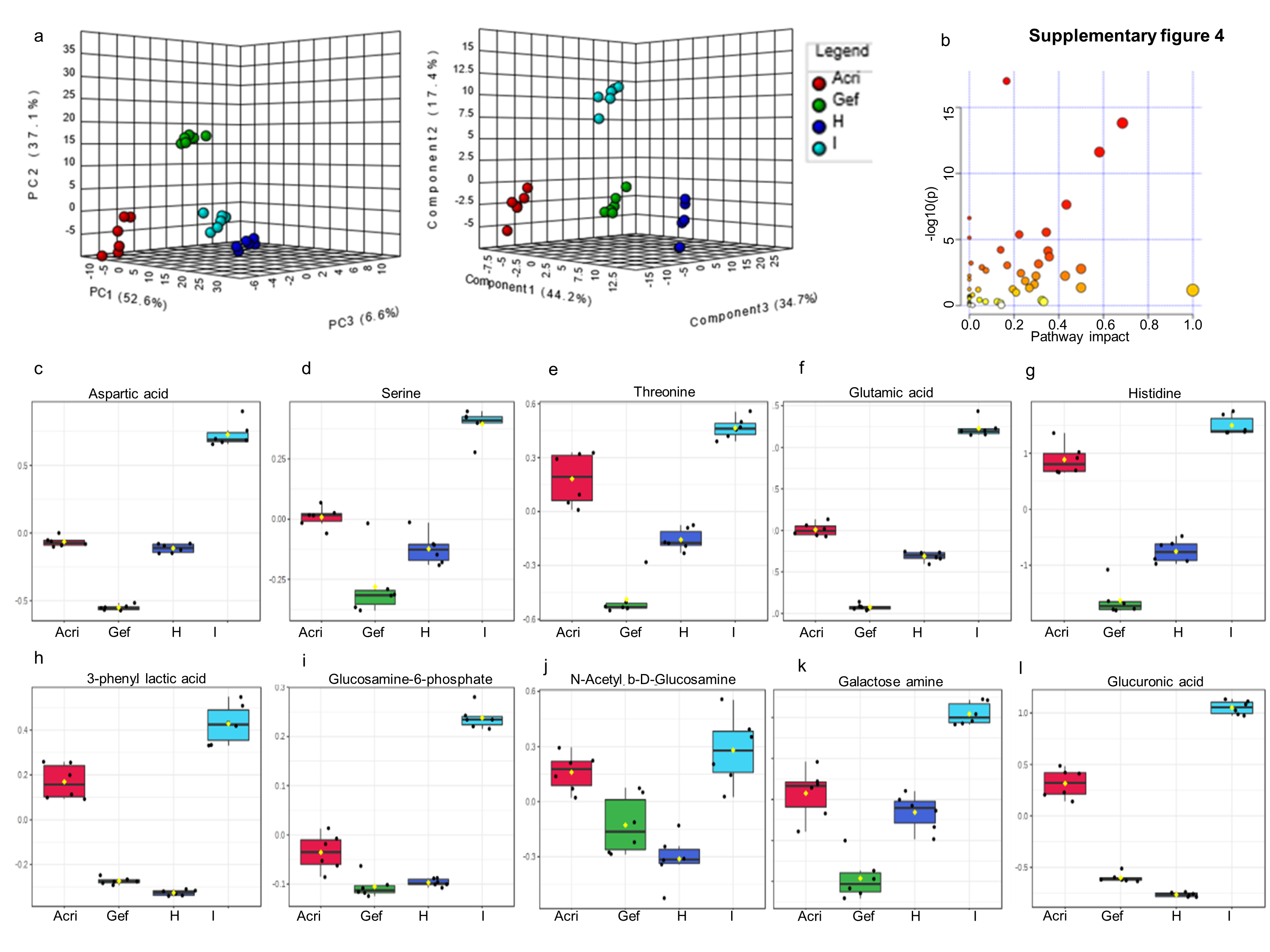

Supplement: Supplementary Figure 1 — Identification of Gefitinib as a potent drug against S. typhi. (A) Cytotoxicity assay of compounds. Left panel shows there was no cytotoxicity or direct killing of Salmonella, the right panel shows no cytotoxicity on THP-1 cells. (B) The bar graph represents % inhibition ± SEM of intracellular replication of S. typhi after the post-exposure of Enzo library compounds (1µM). (C) The number of compounds showing <50%, >50%, >80%, >90% inhibition of intracellular survival on dose dependent kinetics (1µM, 100nM, 10nM). (D) The number of compounds showing invasion inhibition in THP-1 cells from below 50% to above 50%, >80%, >90%. (E, F) Percentage Intra-cellular and invasion inhibition of 3 compounds (dose kinetics assay) in caco2 cells. Data represents mean ± SEM from three replicates and repeated three times each individual experiments. [file Presentation_1.zip › Supplementary 4.TIF]

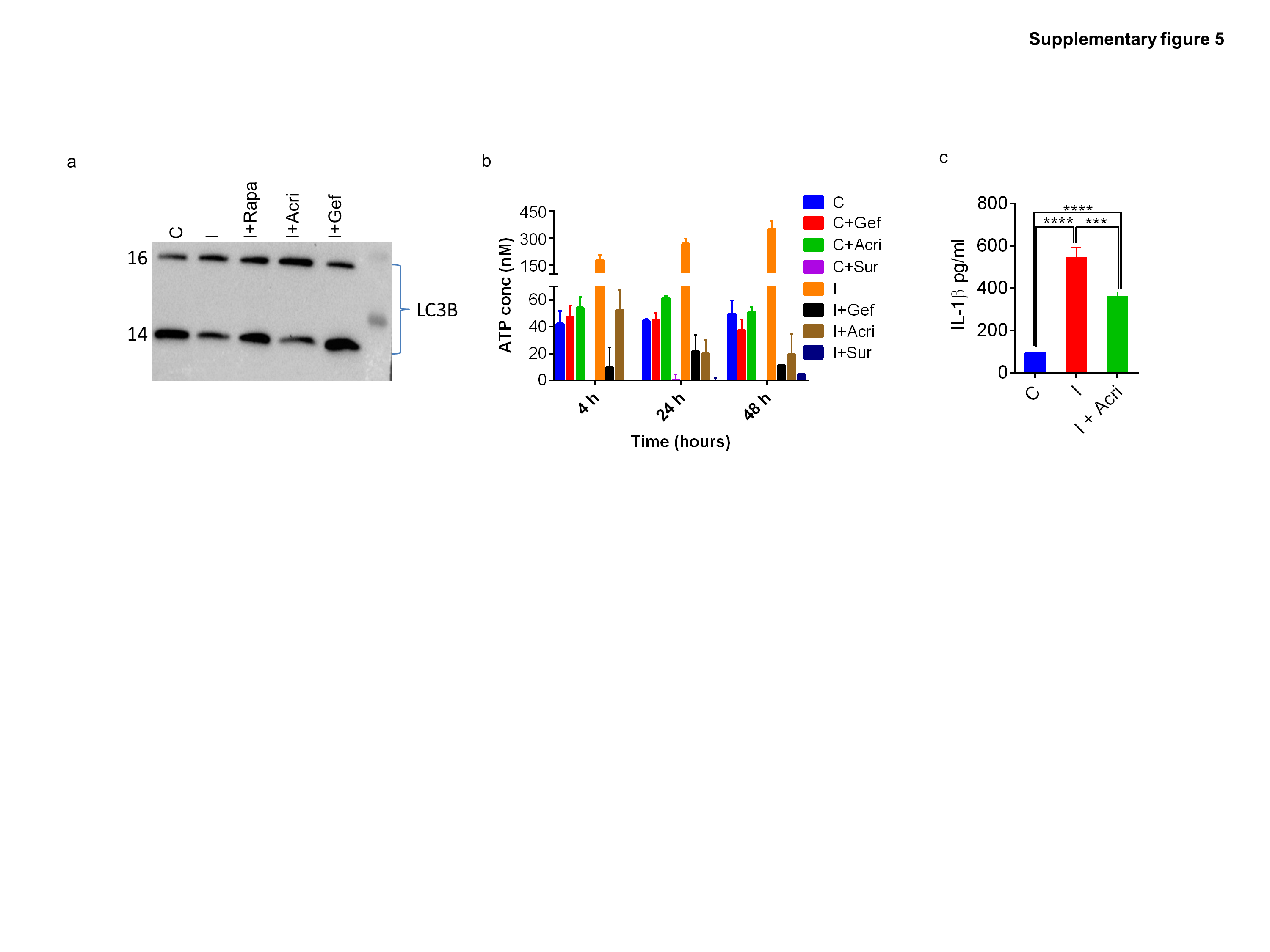

Supplement: Supplementary Figure 1 — Identification of Gefitinib as a potent drug against S. typhi. (A) Cytotoxicity assay of compounds. Left panel shows there was no cytotoxicity or direct killing of Salmonella, the right panel shows no cytotoxicity on THP-1 cells. (B) The bar graph represents % inhibition ± SEM of intracellular replication of S. typhi after the post-exposure of Enzo library compounds (1µM). (C) The number of compounds showing <50%, >50%, >80%, >90% inhibition of intracellular survival on dose dependent kinetics (1µM, 100nM, 10nM). (D) The number of compounds showing invasion inhibition in THP-1 cells from below 50% to above 50%, >80%, >90%. (E, F) Percentage Intra-cellular and invasion inhibition of 3 compounds (dose kinetics assay) in caco2 cells. Data represents mean ± SEM from three replicates and repeated three times each individual experiments. [file Presentation_1.zip › Supplementary 5.TIF]
